# Evinacumab Reduces Triglyceride-Rich Lipoproteins in Patients with Hyperlipidemia: A Post-Hoc Analysis of Three Randomized Clinical Trials

**DOI:** 10.1007/s10557-024-07567-z

**Published:** 2024-03-06

**Authors:** Robert S. Rosenson, Daniel J. Rader, Shazia Ali, Poulabi Banerjee, Jennifer McGinniss, Robert Pordy

**Affiliations:** 1 Metabolism and Lipids Unit, Mount Sinai Heart, Icahn School of Medicine at Mount Sinai, 1 Gustave L. Levy Pl, New York, NY 10029 USA; 2 Department of Genetics and Department of Medicine, Perelman School of Medicine, University of Pennsylvania, Philadelphia, PA 19104 USA; 3 Regeneron Pharmaceuticals, Inc., 777 Old Saw Mill River Road, Tarrytown, New York, NY 10591 USA

**Keywords:** Clinical trials, Evinacumab, Hypercholesterolemia, Hypertriglyceridemia, Triglyceride-rich lipoproteins

## Abstract

**Purpose:**

Natural selection (Mendelian randomization) studies support a causal relationship between elevated triglyceride-rich lipoproteins (TRLs) and atherosclerotic cardiovascular disease (ASCVD). This post-hoc analysis assessed the efficacy of evinacumab in reducing TRLs in patient cohorts from three separate clinical trials with evinacumab.

**Methods:**

Patients with homozygous familial hypercholesterolemia (HoFH) and low-density lipoprotein cholesterol (LDL-C) ≥ 70 mg/dL were enrolled in a phase III trial (R1500-CL-1629; NCT03399786). Patients diagnosed with refractory hypercholesterolemia, with LDL-C ≥ 70 mg/dL or ≥ 100 mg/dL for those with or without ASCVD, respectively, were enrolled in a phase II trial (R1500-CL-1643; NCT03175367). Patients with severe hypertriglyceridemia (fasting TGs ≥ 500 mg/dL) were enrolled in a phase II trial (R1500-HTG-1522; NCT03452228). Patients received evinacumab intravenously (5 or 15 mg/kg) every 4 weeks, or subcutaneously (300 or 450 mg) every week or every 2 weeks. Efficacy outcomes included change in TRLs (calculated as total cholesterol minus high-density lipoprotein cholesterol minus LDL-C) and other lipid parameters from baseline to 12, 16, or 24 weeks for trial 1522, 1643, and 1629, respectively.

**Results:**

At baseline, TRL levels were higher for patients with severe hypertriglyceridemia entering the 1522 trial vs. other cohorts. Reductions in TRLs were observed across all studies with evinacumab, with > 50% reduction from baseline observed at the highest doses evaluated in patients with HoFH or refractory hypercholesterolemia. Within all three trials, evinacumab was generally well tolerated.

**Conclusions:**

Despite limitations in direct comparisons between study groups, these data indicate that TRL levels could be a future target for lipid-lowering therapies.

**Supplementary Information:**

The online version contains supplementary material available at 10.1007/s10557-024-07567-z.

## Introduction

Individuals with elevated levels of low-density lipoprotein cholesterol (LDL-C) have an increased risk for atherosclerotic cardiovascular disease (ASCVD) [1]. For individuals with elevated LDL-C, lipid-lowering therapies (LLTs) are effective in reducing overall cardiovascular risk [2]. However, even if LDL-C treatment thresholds are achieved, a residual risk of ASCVD remains [3]. Triglyceride (TG)-rich lipoproteins (TRLs) are a heterogeneous class of lipoprotein particles that originate from the liver as very-low-density lipoproteins (VLDL) or from the intestine as chylomicrons [4]. Multiple sources of evidence, including Mendelian randomization studies, support a causal relationship between elevated TRL levels and ASCVD, independent of LDL-C levels [4–8].

Angiopoietin-like 3 (ANGPTL3) is an important regulator of lipoprotein metabolism, controlling plasma lipoprotein levels by inhibiting lipoprotein lipase- and endothelial lipase-mediated hydrolysis of TGs and other lipids [9]. ANGPTL3 is an attractive therapeutic target, as ANGPTL3 deficiency increases lipase activity, accelerating the turnover of TRLs [9]. The mechanism for LDL-C lowering through inhibition of ANGPTL3 appears to be independent of the low-density lipoprotein receptor (LDLR) [10]. In the absence of *LDLR*, endothelial lipase de-repression by ANGTPL3 inhibition leads to extensive remodeling of VLDL and the preferential removal of VLDL remnants from circulation via hepatic remnant receptors [10]. This depletes the LDL precursor pool, limits production of LDL particles, and reduces plasma LDL-C levels [10].

The ANGTPL3 monoclonal antibody inhibitor evinacumab has been shown to reduce levels of LDL-C, non-high-density lipoprotein cholesterol (non-HDL-C), apolipoprotein B (ApoB), and apolipoprotein CIII (ApoCIII), all known contributors of risk for ASCVD [11, 12]. Previously, a phase III trial (R1500-CL-1629; NCT03399786) in patients with homozygous familial hypercholesterolemia (HoFH) [11] and a phase II trial (R1500-CL-1643; NCT03175367) in patients with refractory hypercholesterolemia [13] reported significantly reduced LDL-C levels with evinacumab. In addition, a phase II trial (R1500-HTG-1522; NCT03452228) in patients with severe hypertriglyceridemia (sHTG) showed TG reductions in the evinacumab-treated groups vs. placebo [14]. Moreover, in a mechanistic study using ApoB kinetic analysis, evinacumab was associated with an increase in the fractional catabolic rate of intermediate-density lipoprotein ApoB and LDL ApoB, indicating that evinacumab may improve hepatic clearance of TRL remnants from the circulation [15].

Therapies other than evinacumab have also been shown to reduce TRL levels. In a meta-analysis of 15,800 patients with mean baseline TG levels ≥ 177 mg/dL who were treated with statins (rosuvastatin 5 − 40 mg, atorvastatin 10 − 80 mg, and simvastatin 10 − 80 mg), mean percent reductions in TGs across all statins and doses ranged from 15.1% to 31.3% [16]. Moreover, in patients with TG levels as high as 800 mg/dL to 850 mg/dL, statins have been shown to reduce TGs by 40% to 44% in a dose-dependent manner [17]. In the PROMINENT trial comprising patients with type 2 diabetes, mild-to-moderate hypertriglyceridemia, and low HDL and LDL-C levels, pemafibrate, a selective peroxisome proliferator-activated receptor alpha modulator, reduced median fasting TG levels by 31.1%, VLDL cholesterol by 35.0%, remnant cholesterol by 43.6%, and ApoCIII by 27.8% from baseline to 4 months [18]. Omega-3 fatty acids such as eicosapentaenoic acid, docosahexaenoic acid, and icosapent ethyl (ethyl ester of long chain omega-3 fatty acid) can also lower TGs [19, 20]. Among statin-treated patients with hypertriglyceridemia and established cardiovascular disease (or diabetes mellitus and at least one additional risk factor) in the REDUCE-IT trial, a median reduction in TG levels of 18.3% was observed from baseline to 1-year with icosapent ethyl [19]. In the STRENGTH trial comprising statin-treated patients with high cardiovascular risk, hypertriglyceridemia and low high-density lipoprotein cholesterol (HDL-C) levels, an omega-3 carboxylic acid formulation (containing both eicosapentaenoic acid and docosahexaenoic acid) reduced TG levels by 19.0% from baseline to 1-year [20].

Although conventional therapies such as statins and omega-3 fatty acids have been shown to reduce TRLs in patients with dyslipidemia, there is still considerable risk of ASCVD events. Novel therapeutics such as ANGPTL3 inhibitors may offer additional TRL lowering and the potential to provide further cardiovascular benefit.

In this post-hoc analysis, we assessed the efficacy of evinacumab in reducing TRLs in patient cohorts from these three separate clinical trials [11, 13, 14].

## Methods

This post-hoc analysis included data from three separate randomized clinical trials with evinacumab (R1500-CL-1629 [NCT03399786], ELIPSE HoFH; R1500-CL-1643 [NCT03175367]; and R1500-HTG-1522 [NCT03452228]) [11, 13, 14]. For each trial, the clinical study protocol including all amendments were reviewed and approved by the appropriate institutional review board or independent ethics committee at each participating study site. All patients provided written informed consent prior to enrolment. Each trial was reported in accordance with CONSORT reporting guidelines.

The full methodology for the aforementioned trials has been published previously [11, 13, 14]. Briefly, the phase III ELIPSE HoFH trial comprised patients (≥ 12 years of age) with HoFH on stable LLT (± lipoprotein apheresis) and screening LDL-C ≥ 70 mg/dL [11]. The phase II 1643 trial comprised patients (18–80 years of age) with heterozygous familial hypercholesterolemia (HeFH)/non-HeFH patients diagnosed with refractory hypercholesterolemia, with screening LDL-C ≥ 70 mg/dL or ≥ 100 mg/dL for those with or without ASCVD, respectively [13]. Lastly, the phase II 1522 trial comprised patients (18–75 years of age) with sHTG (fasting serum TGs ≥ 500 mg/dL) and with a history of hospitalization for acute pancreatitis. Patients were enrolled into three cohorts based on genotype (Cohort 1, homozygous or compound heterozygous loss-of-function [LOF] lipoprotein lipase [LPL] pathway mutations; Cohort 2, heterozygous LOF LPL pathway mutations; and Cohort 3, without identified LPL pathway mutations). An overview of the study designs for all three clinical trials is shown in Supplementary Fig. 1.

The objective of this post-hoc analysis was to determine the efficacy of evinacumab in reducing TRLs in different patient cohorts from the three separate clinical trials with evinacumab. TRL was calculated as total cholesterol minus HDL-C minus LDL-C. In the 1629 and 1643 trials, LDL-C levels were calculated using the Friedewald equation unless TGs were > 400 mg/dL, when LDL-C was determined via beta-quantification [11, 13]. For the 1522 trial, LDL-C levels were determined via beta-quantification [14].

## Results

Baseline characteristics for the 382 patients included from the three trials are summarized according to trial and treatment arm in Table 1. The mean age within each of the cohort treatment arms ranged from 36.7 years to 55.7 years; the proportion of patients who were White ranged from 72.1% to 95.0%. There were differences in baseline lipid levels across cohorts. Patients with sHTG entering 1522 had higher mean baseline TRLs than those in other cohorts (mean TRLs within treatment arms ranged from 213.9 mg/dL to 249.0 mg/dL for the 1522 cohort and 22.4 mg/dL to 31.7 mg/dL for the 1629 and 1643 cohorts; Table 1).

**Table 1:** Summary of baseline characteristics^a^

Parameter	1629		1643							1522	
	EVIN 15 mg/kg IV Q4W (*n* = 43)	PBO IV Q4W (*n* = 22)	EVIN 450 mg SC QW (*n* = 40)	EVIN 300 mg SC QW (*n* = 42)	EVIN 300 mg SC Q2W (*n* = 39)	PBO SC QW (*n* = 39)	EVIN 15 mg/ kg IV Q4W (*n* = 38)	EVIN 5 mg/kg IV Q4W (*n* = 35)	PBO IV Q4W (*n* = 33)	EVIN 15 mg/kg IV Q4W (*n* = 35)	PBO IV Q4W (*n* = 16)
Age, years, mean (SD)	44.3 (16.8)	36.7 (11.5)	54.5 (15.1)	54.0 (12.2)	55.0 (13.0)	52.4 (12.7)	52.1 (12.1)	55.7 (9.6)	56.2 (10.9)	48.6 (10.2)	46.2 (13.1)
Sex, female, n (%)	24 (55.8)	11 (50.0)	29 (72.5)	23 (54.8)	21 (53.8)	27 (69.2)	19 (50.0)	22 (62.9)	18 (54.5)	17 (48.6)	7 (43.8)
Race, n (%)											
White	31 (72.1)	17 (77.3)	38 (95.0)	39 (92.9)	34 (87.2)	34 (87.2)	35 (92.1)	32 (91.4)	27 (81.8)	29 (82.9)	12 (75.0)
Black	2 (4.7)	0	1 (2.5)	0	0	3 (7.7)	0	0	2 (6.1)	1 (2.9)	0
Asian	6 (14.0)	4 (18.2)	0	0	2 (5.1)	1 (2.6)	0	1 (2.9)	1 (3.0)	5 (14.3)	1 (6.3)
Other or not reported	4 (9.3)	1 (4.5)	1 (2.5)	3 (7.1)	3 (7.7)	1 (2.6)	3 (7.9)	2 (5.7)	3 (9.1)	0	3 (18.8)
BMI, kg/m^2^, mean (SD)	26.1 (5.9)	24.6 (5.7)	27.9 (4.4)	29.3 (4.9)	28.0 (4.4)	29.1 (5.2)	29.3 (4.9)	28.8 (4.6)	28.8 (5.2​)	28.9 (5.1)	28.2 (4.2)
Lipids/lipoproteins, mg/dL^b^											
TRLs	22.4 (13.7)	23.3 (13.0)	26.7 (13.9)	26.0 (11.4)	29.1 (16.1)	26.0 (12.4)	26.3 (11.3)	24.6 (12.6)	31.7 (14.5)	249.0 (154.9)	213.9 (128.6)
TGs	91.0 (65.0:145.0)	103.5 (59.0:182.0)	109.5 (82.0:183.5)	118.5 (83.0:177.0)	128.0 (87.0:167.0)	112.0 (85.0:176.0)	126.5 (89.0:166.0)	102.0 (86.0:156.0)	147.0 (104.0:200.0)	2341.0 (1196.0:3704.3)	1741.5 (993.8:3924.8)
LDL-C	259.5 (172.4)	246.5 (153.7)​	146.3 (84.6)	159.1 (73.0)	136.2 (70.2)	157.8 (92.4)	143.1 (54.4)	146.0 (61.0)	144.5 (46.6)	44.2 (45.5)	37.9 (21.4)
TC	325.6 (170.8)	315.9 (150.4)​	225.5 (86.2)	242.2 (77.3)	217.0 (68.8)	240.1 (91.9)	220.9 (56.8)	228.8 (60.2)	231.6 (50.4)	318.7 (137.5)	270.8 (120.3)
Non-HDL-C	281.9 (172.6)	269.9 (157.8)​	173.0 (84.8)	185.1 (74.8)	165.3 (71.2)	183.9 (92.8)	169.4 (54.2)	170.6 (61.5)	176.2 (48.0)	293.2 (143.7)	251.9 (121.0)
HDL-C	43.6 (14.9)	46.0 (16.1)	52.5 (13.8)	57.0 (22.9)	51.7 (15.5)	56.2 (16.7)	51.5 (17.4)	58.2 (16.8)	55.4 (18.0)	25.5 (23.4)	18.9 (4.0)

^a^Baseline values are presented for the ITT population

^b^Values shown are mean (SD) or median (Q1:Q3)

*BMI* body mass index, *EVIN* evinacumab, *HDL-C* high-density lipoprotein cholesterol, *ITT* intention-to-treat, *IV* intravenous, *LDL-C* low-density lipoprotein cholesterol, *non-HDL-C* non-high-density lipoprotein cholesterol, *PBO* placebo, *Q2W* every 2 weeks, *Q4W* every 4 weeks, *QW* every week, *SC* subcutaneous, *SD* standard deviation, *TC* total cholesterol, *TG* triglyceride, *TRL* triglyceride-rich lipoprotein

**Fig. 1:**
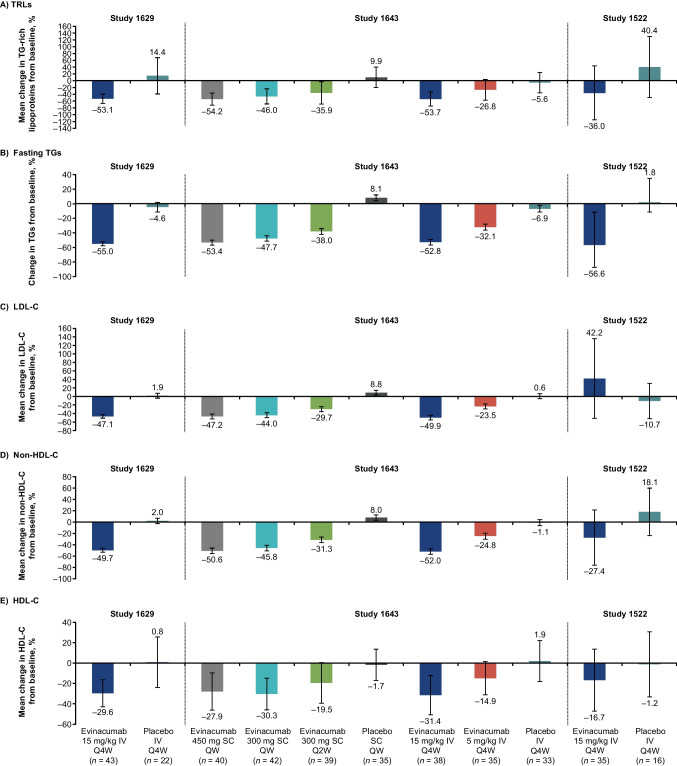
Percent change from baseline in lipids/lipoproteins at study-specific time points according to study and treatment arm^a^. ^a^Percent change from baseline shown at differing timepoints depending on the trial: 1629, Week 24; 1643, Week 16; 1522, Week 12. All analyses are shown for the ITT populations except for HDL-C in 1643 where the results are provided for the safety population. Values shown are mean (SD) except for the following: for 1522, changes in fasting TGs from baseline are shown as median (Q1:Q3); for 1629, changes in fasting TGs are shown as mean (SE); for 1643, changes in fasting TGs, LDL-C, total cholesterol, and HDL-C are shown as mean (SE). *HDL-C* high-density lipoprotein cholesterol, *ITT* intention-to-treat, *IV* intravenous, *LDL-C* low-density lipoprotein cholesterol, *non-HDL-C* non-high-density lipoprotein cholesterol, *QW* every week, *Q2W* every 2 weeks, *Q4W* every 4 weeks, *SD* standard deviation, *SE* standard error, *TG* triglyceride, *TRL* triglyceride-rich lipoprotein

The percent changes from baseline in TRLs and other lipids/lipoproteins at study-specific time points according to study and treatment arm are shown in Fig. 1. In all evinacumab treatment arms, reductions in mean TRLs from baseline were observed, with > 50% reduction from baseline observed at the highest evinacumab doses (mean reduction across all evinacumab treatment arms: 1629 cohort, –53.1%; 1643 cohort, –26.8% to –54.2%; 1522 cohort, –36.0%; Fig. 1A). Mean TRLs was observed to increase from baseline in most placebo treatment groups (1629, + 14.4%; 1643, + 9.9% and –5.6%; 1522, + 40.4%; Fig. 1A). Similarly, reductions in fasting TGs were observed across all evinacumab treatment arms (Fig. 1B). Of note, treatment with evinacumab in patients with sHTG increased levels of LDL-C (42.2%) from baseline, whilst the placebo group saw a 10.7% reduction in LDL-C levels from baseline (Fig. 1C). The increase in LDL-C is consistent with the broader role of evinacumab in TRL metabolism [10], and may be due to the enhanced conversion of very-low density lipoprotein particles to low-density lipoprotein particles, and reduction in ApoCIII, an endogenous lipoprotein lipase inhibitor. Furthermore, levels of non-HDL-C and HDL-C were reduced from baseline in evinacumab-treated patients across the three studies (range –24.8% to –52.0% and –14.9% to –31.4%, respectively; Fig. 1D and 1E).

Overall, safety was consistent across all three trials [11, 13, 14]. In trial 1629, treatment-emergent adverse events (TEAEs) occurred in 66% and 81% of patients in the evinacumab- and placebo-treated groups, respectively; serious adverse events (SAEs) occurred in 5% of the evinacumab group (not considered related to study treatment), with none in the placebo group [11]. In trial 1643, TEAEs ranged from 67 to 84% and 54 to 70% across the evinacumab- and placebo-treated groups, respectively; the incidence of SAEs ranged from 5 to 16% and 3 to 8% across the evinacumab- and placebo-treated groups [13]. In trial 1522, TEAEs occurred in 71.4% and 68.8% of evinacumab- and placebo-treated patients, respectively [14].

## Discussion

In this post-hoc analysis of data from three separate clinical trials, treatment with evinacumab reduced TRL levels and other lipids/lipoproteins in patients with hypercholesterolemia or hypertriglyceridemia [11, 13, 14]. Evinacumab reduced TRL levels by > 50% in the patient cohorts from study 1629 and 1643 who were receiving the highest evinacumab doses. These results are in addition to those previously showing that, for patients with HoFH, evinacumab can effectively reduce LDL-C, non-HDL-C, ApoB, and ApoCIII levels [11]. Therefore, TRL level may be a target for future LLTs.

The importance of TRLs as a future therapeutic target is strengthened by the causal relationship observed between elevated levels of TRLs and an increased risk of ASCVD [4, 5, 7]. There is growing evidence that TRL levels are more predictive of cardiovascular risk than LDL-C [21]. In the primary prevention cohort of the Prevención con Dieta Mediterránea trial, which comprised overweight or obese subjects at high cardiovascular risk, TRL and TG levels were associated with major adverse cardiovascular events; no association was observed with LDL-C [6]. More recently, a multivariate Mendelian randomization analysis that indirectly derived TRL data from 350,110 subjects in the UK Biobank Cohort demonstrated that TRL was associated with an increased risk of coronary heart disease (CHD) independent of ApoB and LDL-C [8]. Moreover, TRL was associated with an increased risk of CHD compared to LDL-C, with odds ratios per 1 mmol/L higher cholesterol of 2.59 and 1.37, respectively [8].

This analysis is not without limitations. The principal limitation of this analysis is that TRL levels were calculated using the formula TRL equals total cholesterol minus HDL-C minus LDL-C, and not measured directly. In study 1629 and study 1643, LDL-C levels for patients with TGs < 400 mg/dL were calculated using the Friedewald equation (LDL-C = [total cholesterol] minus [HDL-C] minus [TGs divided by 5]), which assumes a fixed ratio of 5:1 between TGs and cholesterol in VLDL [22]. Only for patients with TGs > 400 mg/dL was LDL-C measured directly. However, a recent study by Ginsberg and colleagues demonstrated overall good correlation between calculated LDL-C versus LDL-C directly measured via beta-quantification [22]. For beta-quantification derived LDL-C values ≥ 70 mg/dL, there was strong concordance with the LDL-C values calculated using the Friedewald Eq. (97.3%), Martin- Hopkins Eq. (95.3%), and NIH Eq. 2 (96.2%) [22]. Moreover, when TGs were < 150 mg/dL, there were minimal differences between the three formulae and the beta-quantification derived LDL-C values, irrespective of LDL-C level (< 40, < 55, or < 70 mg/dL) [22]. In study 1629 and study 1643 of our analysis, median TG levels were < 150 mg/dL, therefore we do not expect that the use of the Friedewald equation to calculate LDL-C has negatively impacted our calculation of TRLs.

An additional limitation is that the three trials included in our analysis comprise patients with vastly different clinical disorders and eligibility criteria. Moreover, the 1629 trial in patients with HoFH was a phase III trial, whereas the 1643 trial in patients with refractory hypercholesterolemia and 1522 trial in patients with sHTG were both phase II trials. Given these differences, the trial populations cannot be pooled for direct comparison. Furthermore, each trial had a relatively small population and a short treatment duration, preventing the long-term assessment of evinacumab.

## Conclusion

In this post-hoc analysis of three separate clinical trials, treatment with evinacumab in patients with hypercholesterolemia or hypertriglyceridemia showed a reduction from baseline in TRL levels. These data indicate that TRLs could be a future target for lipid-lowering therapies.

## Supplementary Information

Below is the link to the electronic supplementary material.

Supplementary file1 (DOCX 395 KB)

## Data Availability

Qualified researchers may request access to study documents (including the clinical study report, study protocol with any amendments, blank case report form, statistical analysis plan) that support the methods and findings reported in this manuscript. Individual anonymized participant data will be considered for sharing once the product and indication has been approved by major health authorities (e.g., FDA, EMA, PMDA, etc.), if there is legal authority to share the data and there is not a reasonable likelihood of participant re-identification. Submit requests to https://vivli.org/.
