# Supplementary material for: Evinacumab Reduces Triglyceride-Rich Lipoproteins in Patients with Hyperlipidemia: A Post-Hoc Analysis of Three Randomized Clinical Trials
Source: Cardiovasc Drugs Ther. 2024 Mar 6;39(4):925–31. doi: 10.1007/s10557-024-07567-z (PMC12297002; doi:10.1007/s10557-024-07567-z)
Supplement: Supplementary file 1 — Supplementary file1 (DOCX 395 KB) [file 10557_2024_7567_MOESM1_ESM.docx]

Supplementary Figure 1. Study designs for clinical trials with evinacumab: A) 1629, B) 1643, and C) 1522


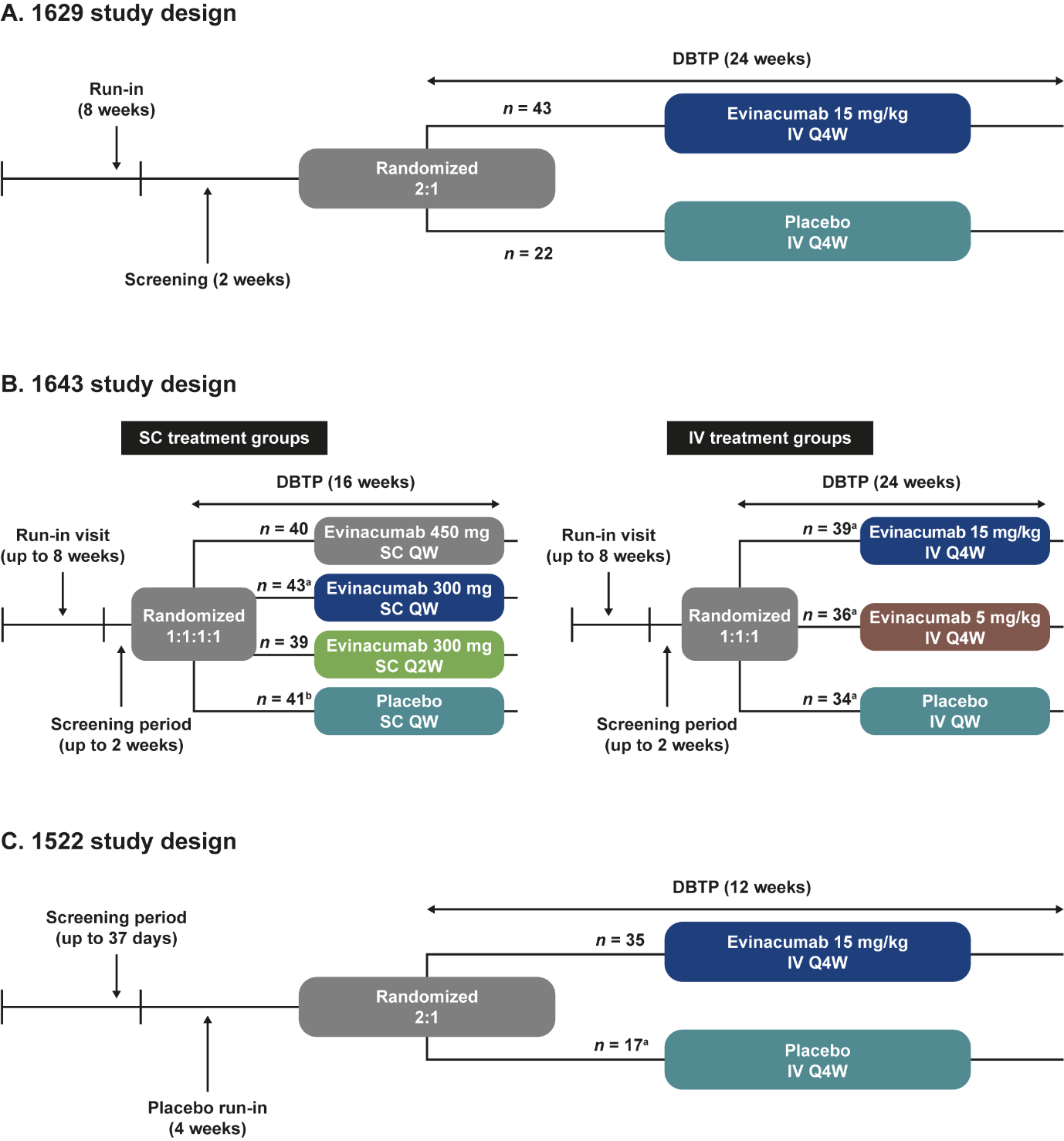


^a^One patient was randomized but not treated.

^b^Two patients were randomized but not treated.

*DBTP* double-blind treatment period, *IV* intravenous, *QW* every week, *Q2W* every 2 weeks, *Q4W* every 4 weeks, *SC* subcutaneous.
